# Hospital Appeals

**Published:** 1918-06-22

**Authors:** 


### Hospital Appeals.

### QUEEN'S HOSPITAL FOR CHILDREN.

Civic patronage has been extended to an appeal for £10,000 which is being made in the interests of the Queen's Hospital for Children, Hackney Road, and, by invitation of the Lord Mayor, a meeting was held recently at the Mansion House. Mr. Alderman Hanson, M.P., presiding, spoke of the excellent work which the institution was doing in the several poor and overcrowded districts which it serves. Last year the receipts available for maintenance purposes were nearly £3,000 less than in 1916, while the expenditure was £2,600 more. The Lord Mayor said it was unthinkable that such a beneficent charity should have to contemplate closing. A sum of about £2,000 has already been secured, including several promises made at the meeting.

### QUEEN MARY'S HOSPITAL FOR THE EAST END.

The Lord Mayor and the Lady Mayoress are interesting themselves in another appeal for £2,500, which is required for the purpose of securing a children's convalescent home for Queen Mary's Hospital, West Ham. The money is being collected by means of a bazaar, which her ladyship opened at the East Ham Town Hall. For the poor little children in a populous district no greater boon, the committee of the hospital states, could be provided than a convalescent home. During the past year 2,026 in-patients, coming from the poorest part of the East End, were cared for.

